# High Expression of Nuclear Factor 90 (NF90) Leads to Mitochondrial Degradation in Skeletal and Cardiac Muscles

**DOI:** 10.1371/journal.pone.0043340

**Published:** 2012-08-17

**Authors:** Takuma Higuchi, Shuji Sakamoto, Yoshihiko Kakinuma, Shoko Kai, Ken-ichi Yagyu, Hiroshi Todaka, Eunsup Chi, Shoshiro Okada, Takako Ujihara, Keiko Morisawa, Masafumi Ono, Yasunori Sugiyama, Waka Ishida, Atsuki Fukushima, Masayuki Tsuda, Yasutoshi Agata, Taketoshi Taniguchi

**Affiliations:** 1 Laboratory of Molecular Biology, Science Research Center, Kochi Medical School, Kochi, Japan; 2 Department of Cardiovascular Control, Kochi Medical School, Kochi, Japan; 3 Department of Pharmacology, Aichi Medical University School of Medicine, Aichi, Japan; 4 The Facility for Radio-isotope Research, Science Research Center, Kochi Medical School, Kochi, Japan; 5 Department of Gastroenterology and Hepatology, Kochi Medical School, Kochi, Japan; 6 Department of Ophthalmology, Kochi Medical School, Kochi, Japan; 7 The Division of Laboratory Animal Science, Science Research Center, Kochi Medical School, Kochi, Japan; 8 Department of Immunology and Cell Biology, Graduate School of Medicine, Kyoto University, Kyoto, Japan; University of Texas Health Science Center at San Antonio, United States of America

## Abstract

While NF90 has been known to participate in transcription, translation and microRNA biogenesis, physiological functions of this protein still remain unclear. To uncover this, we generated transgenic (Tg) mice using NF90 cDNA under the control of β-actin promoter. The NF90 Tg mice exhibited a reduction in body weight compared with wild-type mice, and a robust expression of NF90 was detected in skeletal muscle, heart and eye of the Tg mice. To evaluate the NF90 overexpression-induced physiological changes in the tissues, we performed a number of analyses including CT-analysis and hemodynamic test, revealing that the NF90 Tg mice developed skeletal muscular atrophy and heart failure. To explore causes of the abnormalities in the NF90 Tg mice, we performed histological and biochemical analyses for the skeletal and cardiac muscles of the Tg mice. Surprisingly, these analyses demonstrated that mitochondria in those muscular tissues of the Tg mice were degenerated by autophagy. To gain further insight into the cause for the mitochondrial degeneration, we identified NF90-associated factors by peptide mass fingerprinting. Of note, approximately half of the NF90-associated complexes were ribosome-related proteins. Interestingly, protein synthesis rate was significantly suppressed by high-expression of NF90. These observations suggest that NF90 would negatively regulate the function of ribosome via its interaction with the factors involved in the ribosome function. Furthermore, we found that the translations or protein stabilities of PGC-1 and NRF-1, which are critical transcription factors for expression of mitochondrial genes, were significantly depressed in the skeletal muscles of the NF90 Tg mice. Taken together, these findings suggest that the mitochondrial degeneration engaged in the skeletal muscle atrophy and the heart failure in the NF90 Tg mice may be caused by NF90-induced posttranscriptional repression of transcription factors such as PGC-1 and NRF-1 for regulating nuclear-encoded genes relevant to mitochondrial function.

## Introduction

A group of double-stranded (ds) RNA binding proteins (DRBPs) numbering more than 15 plays key roles in transcription, translation, mRNA processing, transportation, stability and/or editing, and microRNA (miRNA) biogenesis [1], [2]. The DRBPs share dsRNA binding motifs (dsRBM) consisting of 65–68 amino acids that adopt an α-β−β−β−α conformation that confers the ability to bind structured nucleic acids [3], [4]. The dsRBM is evolutionarily conserved from *Escherichia coli* (ribonuclease RNASE-III) through *Saccharomyces cerevisiae* (RNASE-III) and *Drosophila melanogaster* (Staufen), to humans (protein kinase activated by dsRNA (PKR), and TAR RNA binding protein (TRBP) among others) and contributes to binding to DNA or RNA metabolites at the DRBPs involved in multiple cellular events described above.

One such DRBPs is nuclear factor 90 (NF90) (also referred to NFAR1 or DRBP76), which is conserved among human, mouse, rat and *Xenopus* [1]. This protein contains two dsRBMs and a functional nuclear localization signal, and forms a complex with a distinct protein, NF45. The NF90-NF45 complex is predominantly localized in the nucleus. NF90 and NF45 were first isolated as binding factors that associate with a regulatory element of the interleukin (IL)-2 promoter, known as an antigen receptor response element, in an activated Jurkat T-cell line [5]. Independently, we identified NF90 as a nuclear factor that recognizes a unique palindromic sequence in the DNase I-hypersensitive site, which is a transcriptional regulatory region of the HLA-DRα gene, in monocytic leukaemia THP-1 cells [6]. Recently it has also been reported that NF90 and NF45 function as novel regulators of IL-13 transcription response to T cell activation [7]. NF90 is also known to bind to a minihelix RNA derived from adenovirus VA RNA [8]. Interestingly, the secondary structures of both the palindromic sequence within the HLA-DRα gene and the minihelix RNA are predicted to form a small double-stranded structure similar to the structures of miRNA precursors (primary (pri)- and precursor (pre)-miRNA) which are intermediates of miRNA biogenesis. Based on this hypothesis, we examined the role of NF90 in miRNA biogenesis. We determined that the NF90 and NF45 complex negatively regulates the pri-miRNA processing step [9]. In addition to roles in transcription and miRNA biogenesis, it has been reported that NF90 participates in RNA splicing [10], mRNA stability and/or transportation [11]–[13], translation [14] and regulation of virus replication [15].

Although the observations described above imply that NF90 is a multi-functional DRBP, physiological functions of this protein remain obscure. To uncover this, we have generated transgenic (Tg) mice that overexpress NF90 and have investigated the functions of this protein *in vivo*.

## Results

### Generation of NF90 Transgenic (Tg) Mice, and Body Growth Rates of Wild-type (WT) and NF90 Tg Mice

**Figure 1:**
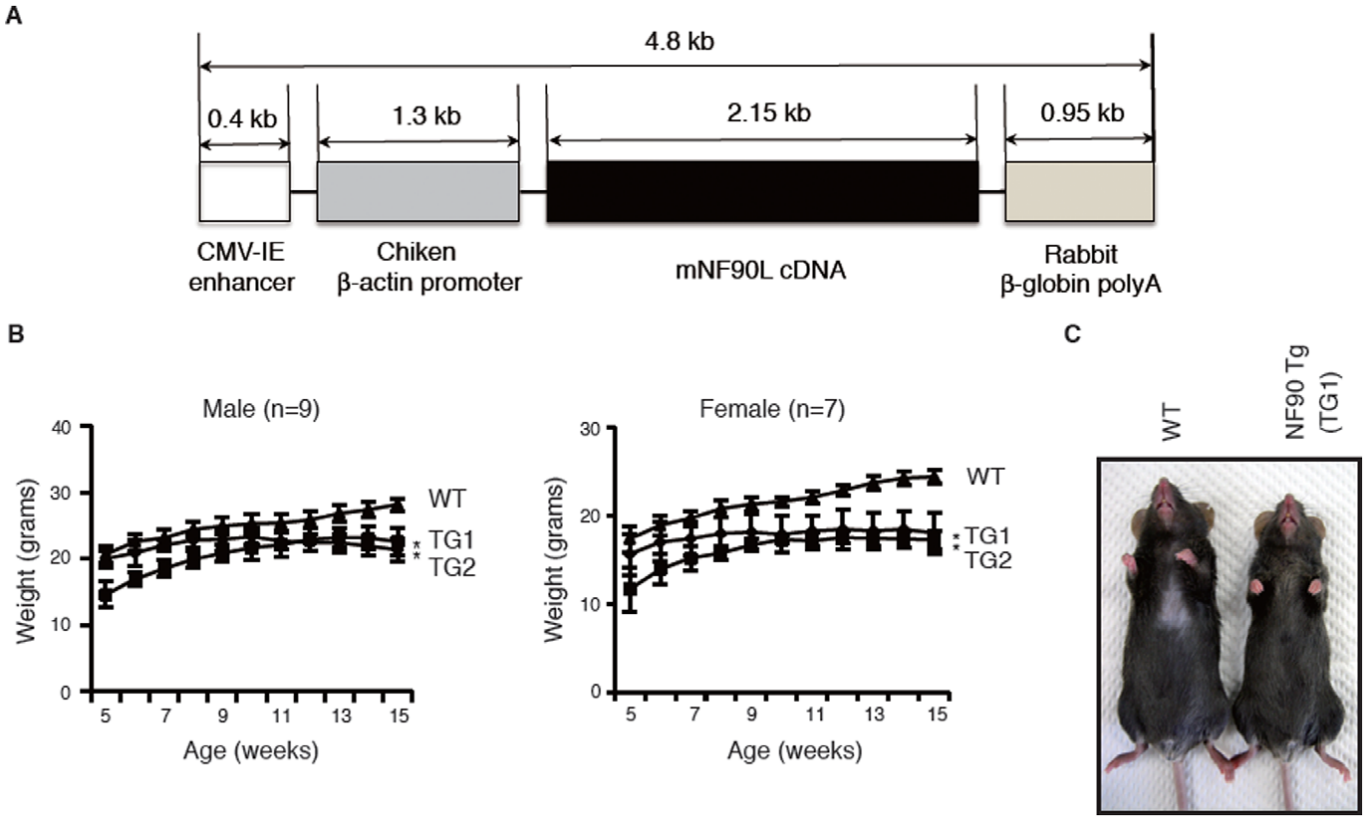
Structure of the NF90 transgene and body growth rates of wild-type (WT) and NF90 transgenic (Tg) mice. (A) NF90 transgene construct used to generate transgenic mice. (B) Growth curves of WT and two lines NF90 Tg male (n = 9) and female (n = 7) mice (lines TG1 and TG2) from the age of 5 weeks through 15 weeks. Data are expressed as means ± SD. *, p < 0.0001 relative to WT by a two-tailed Student’s t test. (C) Photograph of whole body from WT and NF90 Tg male mice (line TG1) at 12 weeks of age.

To explore the physiological functions of NF90, we set out to generate transgenic mice. We initially attempted to overexpress this protein in many tissues, and with this goal in mind we used the chicken β-actin promoter and the cytomegalovirus enhancer [16] for the transgenic construct (Figure 1A), and two transgenic lines (TG1 and TG2) were finally established. To our surprise, however, the transgene was preferentially expressed in heart, skeletal muscle and eyes of both lines, and a slightly elevated level of expression was detected in other tissues (Figure S1A and S1B). The expression levels of NF90 in heart and skeletal muscles between the two lines were largely identical. Its predominant expression in skeletal and cardiac muscles of the TG mice was also confirmed by immunohistochemistry analysis of NF90 (Figure S2, compare II and VI with IV and VIII). Because at least two independent transgenic lines showed a similar pattern, it suggests that this pattern of expression is not due to the position of the transgene insertion. Of note, the NF90 Tg mice showed a reduction in body weight and size compared with WT mice over 10 weeks of age (Figure 1B and 1C). The reduction level was comparable between male and female mice (Figure 1B).

### NF90 Tg Mice Display Muscular Atrophy and Heart Failure

**Figure 2:**
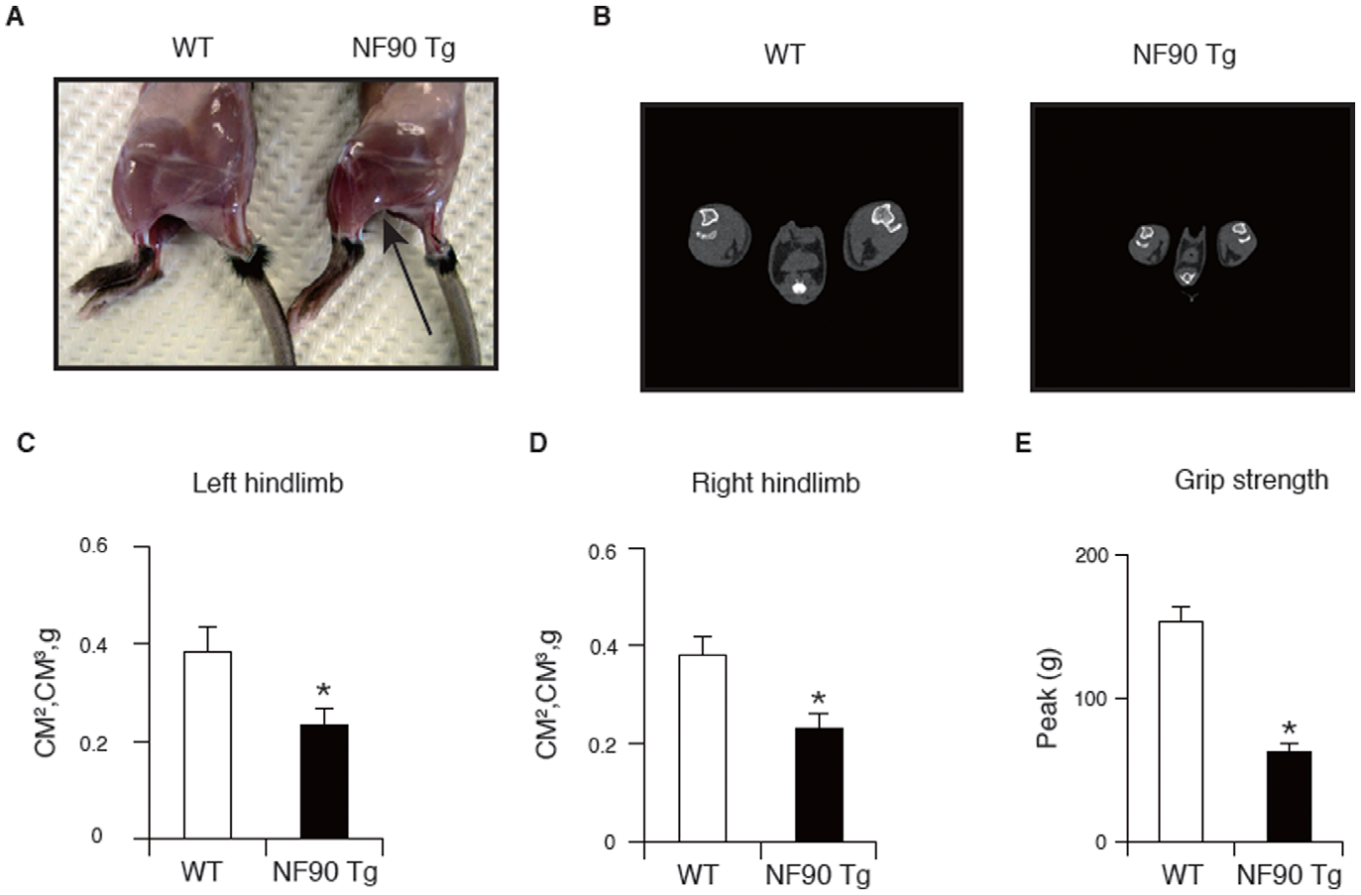
NF90 Tg mice display muscular atrophy. (A) Lateral view of hindlimbs from WT and NF90 Tg mice (line TG1) at 12 weeks of age. An arrow indicates muscular atrophy in the NF90 Tg mice. (B) Axial computed tomography images of the center of distal hindlimbs in WT and NF90 Tg mice (line TG1) at 12 weeks of age. (C and D) Comparison of volumes of triceps surae muscles from left (C) and right (D) hindlimbs between WT and NF90 Tg mice (line TG1) at 12 weeks of age. The muscle volumes were measured by using X-ray computed tomography. Data are expressed as means ± SD (n = 5). *, p < 0.005 relative to WT by a two-tailed Student’s t test. (E) Peak force measurements (g) of grip strength of WT and NF90 Tg mice (line TG1) at 16 weeks of age. Data are expressed as means ± SD (n = 5). *, p < 0.001 relative to WT by a two-tailed Student’s t test.

To verify the influence of high-expression of NF90 on the physiological functions of the skeletal and cardiac muscles of the Tg mice, we performed computed tomographic (CT) analysis to evaluate muscle volume, a grip strength test for the measurement of muscle force and hemodynamic analysis by measuring blood pressure (BP) and heart rate (HR). As shown in Figure 2A, the quadriceps and the gastrocnemius muscles of the NF90 Tg mice at 12 weeks of age become thinner than those of WT at the same age. X-ray CT analysis revealed that the volumes of the gastrocnemius muscle of both hindlimbs of the NF90 Tg mice were significantly decreased compared with those of WT mice (Figure 2B, 2C and 2D; *P* < 0.005). An automated grip strength meter showed that the grip strength of the limbs of the NF90 Tg mice was significantly reduced compared with that of WT mice (Figure 2E; *P* < 0.001). These results indicated that the NF90 Tg mice display skeletal muscle atrophy.

**Figure 3:**
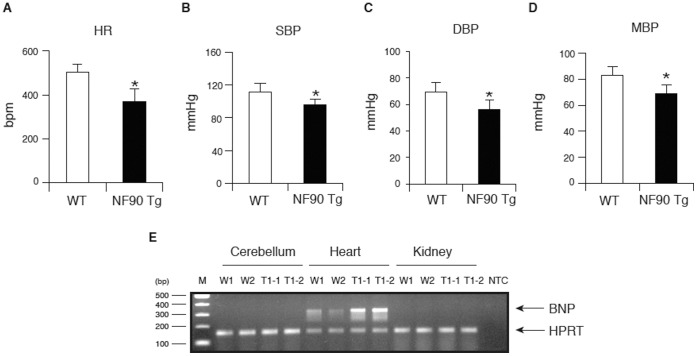
NF90 Tg mice display heart failure. (A to E) Measurement of heart rate, blood pressure (BP) and brain natriuretic peptide (BNP) of WT and NF90 Tg mice (line TG1) at 14 weeks to 19 weeks of age. Heart rate and BP of mice were measured by a programmable sphygmomanometer using tail-cuff method. (A) HR, heart rate; (B) SBP, systolic blood pressure; (C) DBP, diastolic blood pressure; (D) MBP, mean blood pressure. All data are expressed as means ± SD (n = 7 per group). *, p < 0.01 relative to WT by a two-tailed Student’s t test. (E) The expression of BNP in NF90 Tg mice. Total RNAs isolated from cerebellum, heart and kidney of WT and NF90 Tg mice (line TG1) were analyzed by RT-PCR with specific primers for BNP or HPRT. HPRT was used as an internal control. W-1 and W-2, wild-type; T-1 and -2, NF90 Tg mice (line TG1).

In addition, a hemodynamic assessment using a tail-cuff method showed that the HR and BPs in the NF90 Tg mice were significantly lower than those in WT mice (Figure 3A, 3B, 3C and 3D; *P* < 0.01). We also measured the expression of brain natriuretic peptide (BNP) which is known as a marker of heart failure. The results indicate that the gene expression level of BNP was clearly increased in the hearts of NF90 Tg mice compared with WT mice, but was not detected in the cerebellum and kidney (Figure 3E). In addition, the higher level of blood noradrenaline as shown in Figure S3 suggested that the NF90 Tg mice are in heart failure.

Collectively, these observations together with Figure 1B and 1C indicate that the skeletal muscular atrophy and the heart failure would influence a reduction in body weight in the NF90 Tg mice.

### Skeletal and Cardiac Muscles of NF90 Tg Mice Exhibit Mitochondrial Degeneration

**Figure 4:**
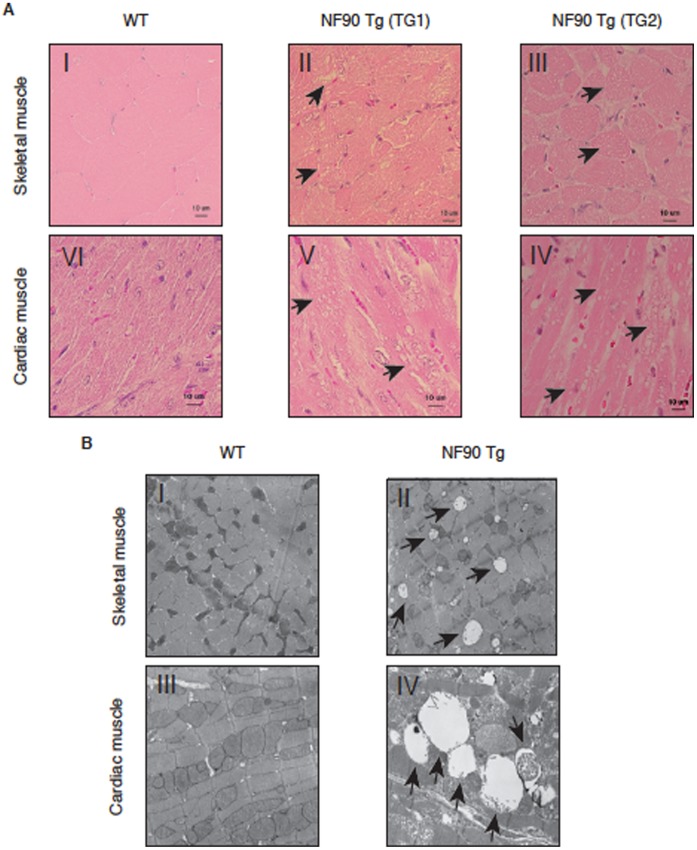
Skeletal and cardiac muscles of NF90 Tg mice exhibit mitochondrial degradation. (A) Haematoxylin and eosin (HE)-stained sections of skeletal (I to III) and cardiac (VI to IV) muscles from WT (I and VI) and NF90 Tg mice (line TG1: II and V, line TG2: III and IV) at 15 to 17 weeks of age. Arrows highlight vacuolations. Scale bars show 10 mm at the inset. (B) Transmission electron microscopy analysis of skeletal (I and II) and cardiac (III and IV) muscles from WT (I and III) and NF90 Tg mice (II: line TG2, IV: line TG1) at 13 to 17 weeks of age. Arrows indicate degenerating mitochondria.

To gain insight into the cause for the skeletal muscular atrophy and the heart failure in the NF90 Tg mice, we performed histological analysis using haematoxylin and eosin (H&E) staining of skeletal and cardiac muscles of WT and NF90 Tg mice. The analysis indicates that the skeletal and cardiac muscles of the NF90 Tg mice have a large number of vacuoles (Figure 4A, arrows). To investigate these vacuolations, we carried out an electron microscopy analysis. As shown in Figure 4B, numerous mitochondria in the skeletal and cardiac muscles of the Tg mice are degenerated and became vacuolated (Figure 4B, arrows in panels II and IV). These results demonstrate that the vacuoles in the muscle tissues of the NF90 Tg mice were caused by mitochondrial degeneration. Further earlier analysis with H&E staining of the cardiac muscles reveals that the vacuolation occurs in the NF90 Tg mice at later than 9 weeks of age (Figure S4, arrows in panels IX and X). Concomitantly we examined the expression of NF90 protein in the cardiac muscles of the Tg mice from 6 to 10 weeks and 18 weeks of age. These data show that NF90 was already expressed in the NF90 Tg mice over 6 weeks of age (Figure S5), indicating that the vacuolation of mitochondria follows NF90 expression. These findings suggest that a reduction in body weight of NF90 Tg mice over 10 weeks of age caused by the skeletal muscle atrophy and the heart failure tightly correlates with the mitochondrial degeneration of the skeletal and cardiac muscles.

**Figure 5:**
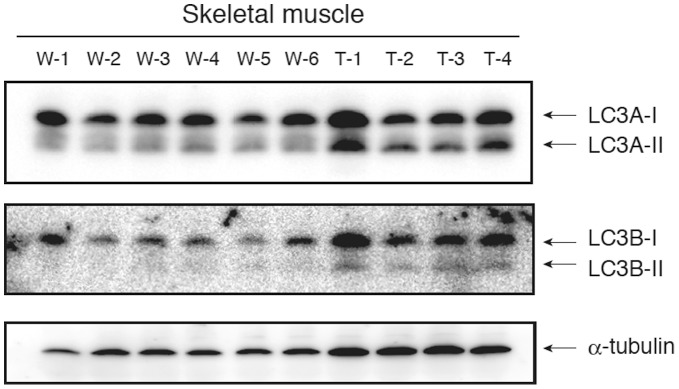
Autophagy is induced in the skeletal muscles of the NF90 Tg mice. Immunoblot analysis of LC3A and LC3B in tissue extractions of skeletal muscles of WT (n = 6) and NF90 Tg mice (line TG1) (n = 4). Anti-α-tubulin was used as loading control. W-1 to -6, WT; T-1 to -4, NF90 Tg mice (line TG1).

Degradation of cytoplasmic components including mitochondria is known to be achieved by autophagy. Particularly, skeletal and cardiac muscles are the most sensitive tissues to in vivo autophagic degradation frequently enclosed mitochondria [17]. Therefore, we examined whether the mitochondrial vacuolation of the skeletal and cardiac muscles in the NF90 Tg mice is due to autophagocytosis. LC3, which is a mammalian homologue of yeast autophagy-related gene (Atg) 8, serves as a molecular marker for autophagosomes [18]. LC3 is present in two different forms referred to as LC3-I and LC3-II. When autophagy occurs, LC3-I is converted to the second form, LC3-II, which associates with autophagosomes [18]. LC3 is also expressed as 3 splice variants (LC3A, LC3B and LC3C). Because LC3A and B are distributed abundant in mammalian skeletal muscles [19], [20], we measured the expressions of those proteins in the skeletal muscles of WT and NF90 Tg mice. The results indicated that the amounts of LC3A/B-II are markedly higher in the NF90 Tg mice than in WT mice (Figure 5). On the other hands, we also tested whether apoptosis is induced in the skeletal muscles of the NF90 Tg mice. As shown in Figure S6, cleaved caspase-3 and -6, which are key modulators of the apoptotic pathway, were not found in the NF90 Tg mice. These findings implies that the degradation of mitochondria in the NF90 Tg mice is due to autophagocytosis, not apoptotic cell death.

### NF90 Represses Protein Synthesis

**Figure 6:**
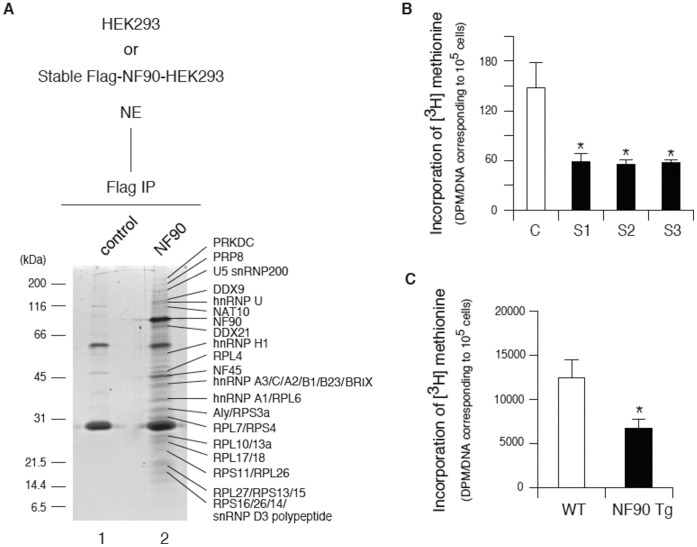
NF90 represses protein synthesis rate. (A) Isolation of NF90-associated complexes. The resulting peptide mass fingerprints were compared to those in a data base, and the identity of each protein is shown to the right of the gel. The molecular markers are indicated on the left in kilodaltons (kDa). (B and C) Protein synthesis rate in untransfected (C) and Flag-NF90-HEK 293 stable cells (S1, S2, and S3) (B) and primary cells of skeletal muscles from WT (n = 2) and NF90 Tg mice (line TG1) (n = 3) at 11 to 13 weeks of age (C). Protein synthesis was measured in cells incubated in medium containing [^3^H]methionine. The DNA amounts in the cells used in this assay were quantitated by the DABA method for normalization of the data. Data are expressed as [^3^H]methionine incorporated (DPM/DNA amount corresponding to 10^5^ cells) and are expressed as means ± SD of three independent experiments. *, p < 0.01 relative to control by a two-tailed Student’s t test.

To investigate the cause for the mitochondrial degradation in the NF90 Tg mice, we isolate NF90-associated complexes from cultured cells overexpressing NF90. The NF90-associated complexes were purified from HEK293-derived stable cell lines expressing Flag-tagged NF90 by immunoprecipitation using anti-Flag-conjugated beads followed by SDS-PAGE analysis. The results indicated that the NF90-associated complexes contained more than 20 proteins spanning a broad molecular weight range (Figure 6A, lane 2). The major protein components of the immunoprecipitated NF90-associated proteins were investigated by mass spectrometry. The identification of the complexes was carried out for individual bands excised from SDS-PAGE gels using in-gel digestion, followed by MALDI-TOF-MS/MS peptide mass fingerprinting. This analysis yielded 17 ribosomal proteins and 17 non-ribosomal proteins (Figure 6A and Table S1). Almost all of the non-ribosomal proteins were known to be involved in pre-mRNA splicing, mRNA transport and ribosome biogenesis (Table S1). In particular, it is noteworthy that half of the NF90-associated complexes were ribosomal proteins. These results indicate that NF90 may be engaged in the function of ribosomes. To verify this possibility, we measured the protein synthesis rate in the Flag-NF90-HEK 293 stable cells and primary cells from the skeletal muscle of the NF90 Tg mice by the incorporation of [^3^H]methionine into proteins. Immunoblot analysis confirmed the overexpression of NF90 in the primary cells of the Tg mice (Figure S7). As shown in Figure 6B and 6C, the protein synthesis rates in the stable cell lines overexpressing NF90 and the primary cells from skeletal muscle of the NF90 Tg mice were significantly reduced compared with those of the control cells. These results suggest that the NF90 would negatively regulate the function of ribosome via its interaction with the ribosomal proteins and/or non-ribosomal proteins involved in ribosomal biogenesis. Therefore, the reduction in nuclear DNA-derived mitochondria-related proteins by NF90 may influence the mitochondrial degradations in the NF90 Tg mice.

### The Protein Level of PGC-1 is Reduced in the Skeletal Muscles of the NF90 Tg Mice

**Figure 7:**
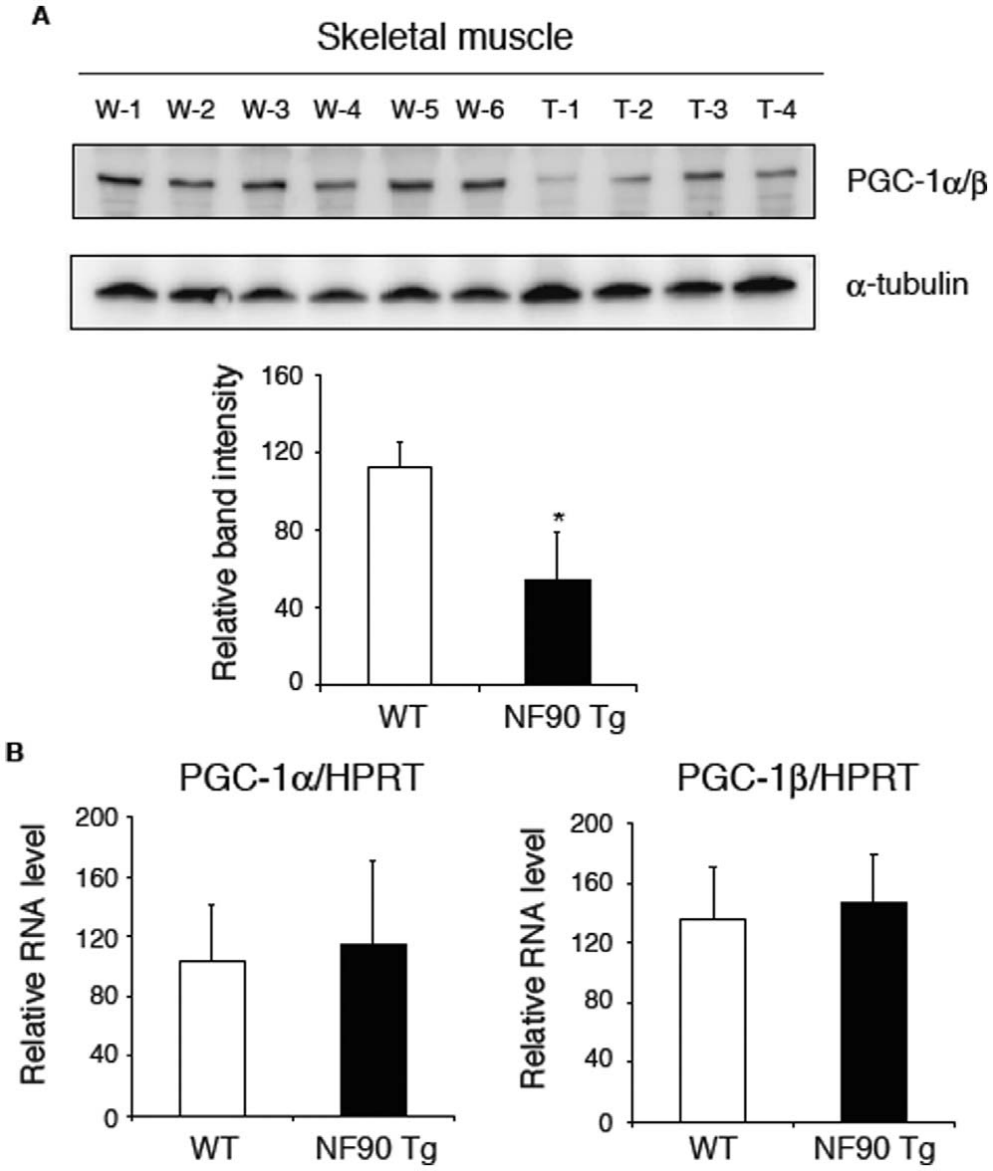
The translation of PGC-1 is depressed in the skeletal muscles of the NF90 Tg mice. (A) Immunoblot analysis of PGC-1 in tissue extractions of skeletal muscles of WT (n = 6) and NF90 Tg mice (line TG1) (n = 4). Anti-α-tubulin was used as loading control. W-1 to -6, WT; T-1 to -4, NF90 Tg mice (line TG1). Intensities of specific bands in imunoblotting analysis were measured with a densitometer and are presented as a graph. Data are expressed as means ± SD. *, p < 0.01 relative to WT by a two-tailed Student’s t test. (B) RNAs isolated from skeletal muscles of WT (n = 4) and NF90 Tg mice (line TG1) (n = 3) were analyzed for expressions of PGC-1α and PGC-1β by RT-qPCR. HPRT was used as an internal control and for normalization of the data. Data are expressed as means ± SD.

**Figure 8:**
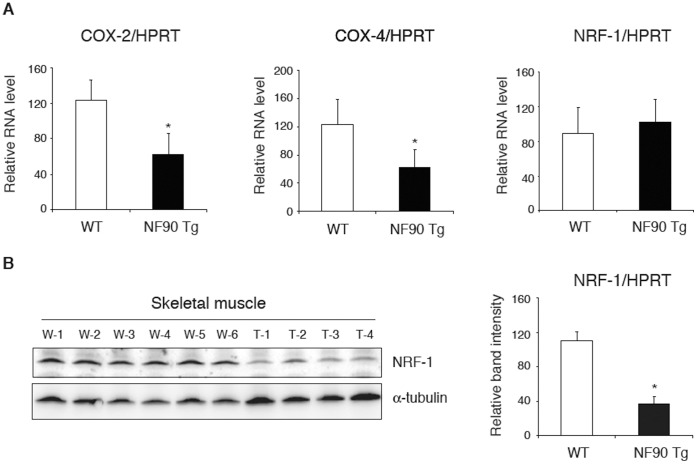
Expression analysis of mitochondria-related proteins, COX-2, COX-4 and NRF-1 in the skeletal muscles of WT and NF90 Tg mice. (A) RNAs isolated from skeletal muscles of WT (n = 6) and NF90 Tg mice (line TG1) (n = 4) were analyzed for expressions of COX-2, COX-4 and NRF-1 by RT-qPCR. HPRT was used as an internal control and for normalization of the data. Data are expressed as means ± SD. *, p < 0.01 relative to WT by a two-tailed Student’s t test. (B) Immunoblot analysis of NRF-1 in tissue extractions of skeletal muscles of WT (n = 6) and NF90 Tg mice (line TG1) (n = 4). Anti-α-tubulin was used as loading control. W-1 to -6, WT; T1-1 to -4, NF90 Tg mice (line TG1). Intensities of specific bands in imunoblotting analysis were measured with a densitometer and are presented as a graph. Data are expressed as means ± SD. *, p < 0.01 relative to WT by a two-tailed Student’s t test.

In nuclear-encoded mitochondria-related proteins, PGC-1 is known to be a key molecule for mitochondrial biogenesis [21], [22]. Therefore, this prompted us to examine the expression of PGC-1 in the muscular tissues with vacuolar degeneration of mitochondria in the NF90 Tg mice. As shown in Figure 7, we found that the PGC-1α/β expressions at protein level were significantly decreased in the skeletal muscles of the NF90 Tg mice compared with those of WT mice (Figure 7A), whereas there were no difference in the mRNA expression levels of PGC-1α and PGC-1β between WT and the NF90 Tg mice (Figure 7B). These results indicate that high-expression of NF90 negatively acts on the translations or protein-stabilities of PGC-1α/β. We next examined whether expression levels of mitochondrial genes including cytochrome c oxidase (COX) -2, COX-4 and nuclear respiratory factor-1 (NRF-1), which are downstream targets of PGC-1, are altered in the skeletal muscles of the NF90 Tg mice. As expected, the expression of COX-2, which is a mitochondrial DNA (mtDNA)-encoded protein, was significantly decreased in the NF90 Tg mice bearing mitochondrial disruption in the skeletal muscles (Figure 8A). The level of COX-4, which is a nuclear-encoded mitochondrial respiratory protein, was also significantly reduced in the NF90 Tg mice, while there was no difference in the mRNA level of NRF-1, which is a transcription factor for regulating nuclear-encoded genes relevant to mitochondrial function, between WT and the NF90 Tg mice (Figure 8A). However, we observed that the protein level of NRF-1 is significantly diminished in the muscles of the NF90 Tg mice (Figure 8B). PGC-1α coactivates the transcriptional function of NRF-1 on the nuclear-encoded subunits of mitochondrial respiratory chain including COX-4 [22]. Therefore, these findings suggest that the fall in translational levels or protein-stabilities of nuclear-encoded mitochondria-related transcription factors such as PGC-1α and NRF-1 causes the downregulation of the nuclear-encoded mitochondrial genes, resulting in the vacuolar degradation of mitochondria follows a reduction of COX-2 in the muscular tissues of the NF90 Tg mice.

## Discussion

The most striking feature of the NF90 transgenic mice which were used in this study was a reduction in body weight and size at over 10 weeks of age (Figure 1B and 1C). Further analysis revealed that the NF90 Tg mice exhibited skeletal muscular atrophy and heart failure accompanied with mitochondrial vacuolation which is caused by autophagocytosis (Figure 2, 3, 4, 5 and S3). As mentioned earlier, the NF90 protein has two dsRBMs which are responsible for protein-protein interactions [23] as well as binding to RNA. Indeed, it has been reported that NF90 interacts with various proteins including PKR [24], DNA-PKcs [25], Ku [26] and protein-arginine methyltransferase I [27]. To gain insight into the cause for the muscular abnormality of the NF90 Tg mice, we comprehensively identified components of the NF90-associated complex by peptide mass fingerprinting and found that approximately half of the NF90-interacting factors were ribosomal protein and ribosome biogenesis factors (Figure 6A and Table S1). Thereafter, we sought to investigate the influence of NF90 on ribosome function. As a result, we found that overexpression of NF90 causes a significant reduction in the protein synthesis rate (Figure 6B and 6C). This result is consistent with a previous study demonstrating that the protein synthesis rate and the amount of polysome were elevated in HeLa cells depleted of NF90/110 [28]. In mitochondria, the coding capacity of mammalian mtDNA is limited to 13 oxidative phosphorylation polypeptides, 22 tRNAs, and 2 rRNA. Thus, the vast majority of mitochondrial proteins are nuclear DNA-encoded. Biogenesis and function of mitochondria require, therefore, the tightly coordinated nuclear DNA and mtDNA-derived protein synthesis. High expression of NF90 is assumed to suppress the translations of nuclear DNA-derived proteins, because NF90 localizes predominantly to the nucleus [29]. Therefore, it is possible that the mitochondrial vacuolations in the skeletal and cardiac muscles of the NF90 Tg mice are caused by the reduction in the translations of nuclear DNA-encoded mitochondria-related proteins by the overexpression of the NF90 protein.

In the nuclear DNA-encoded mitochondria-related proteins, PGC-1 is well known to play a key role in mitochondrial biogenesis. PGC-1 was originally identified as a peroxisome proliferator-activated receptor-γ (PPARγ)-interacting protein from brown fat [21]. Since then, it has become apparent that PGC-1 can bind to, and coactivate many other transcription factors as well as most members of the nuclear hormone receptors. Among the factors that PGC-1 coactivate, the transcription factors, NRF-1 and NRF-2, and the nuclear hormone receptors such as estrogen-related receptor α (ERRα), are known to directly regulate the expression of certain nuclear-encoded mitochondrial genes. NRF-1, NRF-2 and ERRα coactivated by PGC-1 have been linked to the transcriptional control of mitochondrial genes encoded in the nucleus, including COX-4 and ATP synthase β-subunit [22], [30]. Mitochondrial transcription factor A (mtTFA), which is a mitochondrial matrix protein essential for the replication and transcription of mitochondrial DNA, is also activated at the transcriptional level by NRF-1 and -2 and ERRα together with PGC-1 [22], [30]. Notably, we have found that the expressions of PGC-1α/β and NRF-1 at the protein level significantly diminish in the skeletal muscles of the NF90 Tg mice (Figure 7A and Figure 8B). Further, the mRNA levels of the nuclear-encoded mitochondrial respiratory protein, COX-4, and the mtDNA-encoded respiratory chain subunit, COX-2, were also significantly reduced in the muscles of the NF90 Tg mice (Figure 8A). These observations suggest that the fall in the PGC-1α/β and NRF-1 protein levels may deeply correlate with the mitochondrial degeneration in the muscular tissues of the NF90 Tg mice, leading to the skeletal muscle atrophy and the heart failure in the Tg mice.

Mice with targeted disruption of NF90 were previously generated and the phenotype of the mice has been investigated [31]. Intriguingly, the NF90 null mice also exhibit the skeletal muscular abnormalities with the reduction in muscle fibers caused by developmentally retardation of muscular differentiation, and the retardation of the muscular differentiation is thought to be due to down-regulations of myogenic regulatory factors (MRFs), MyoD, Myogenin, and p21 [31]. This study further demonstrates that NF90 interacts with 3’-UTRs of p21 and MyoD mRNA sequences which are predicted to adopt secondary structures including regions of double-stranded RNA and hairpin loops, suggesting that NF90 may participate in post-transcriptional stabilization of the MRFs mRNAs via the binding of NF90 to the secondary structures of the 3’-UTRs [31]. In the present study, we demonstrate that the protein synthesis rate is suppressed by the overexpression of NF90 (Figure 6 B and C), while the protein level of α-tubulin was not reduced in the NF90 Tg mice compared with WT mice (Figure 5, 7A, 8B and S6). On the other hand, the expressions of PGC-1α/β and NRF-1 proteins are significantly decreased in the skeletal muscles of the NF90 Tg mice (Figure 7A and 8B). These findings suggest that the secondary structural specificity of NF90 to mRNAs may play a crucial role in NF90-induce translational repression. Elucidation of this issue will be of interest but will require extensive work in the future.

## Materials and Methods

Sequences of all the oligonucleotides are listed in Table S2 of the supplemental information.

### Generation of Transgenic Mice and Genotyping

To generate NF90 transgenic mice, mouse NF90 (mNF90) cDNA was amplified from a mouse leukaemic monocyte macrophage cell line (RAW cells) (ATCC) by RT-PCR. The cDNA fragment was subcloned into the XhoI site of the pCAGGS vector provided by Dr. Jun-ichi Miyazaki, Osaka University, Osaka [16]. After digestion with SalI and AvrII, the fragment carrying the CMV-IE enhancer, chicken β-actin promoter, mNF90 cDNA and rabbit β-globin polyA was used for microinjection into fertilized eggs recovered from C57BL/6CrSlc females crossing with C57BL/6CrSlc males at Japan SLC inc. (Hamamatsu, Japan). Transgenic mice were identified by PCR analysis of tail genomic DNA. All animal experiments were approved by the Division of Laboratory Animal Science, Science Research Center, Kochi University.

### Computed Tomographic Analysis of Muscle Volume

The muscle volume of the distal hindlimb was measured by a Latheta X-ray computed tomograph (Hitachi Aloka Medical, Ltd., Japan) according to the manufacturer’s protocol.

### Grip Strength Test

Grip strength was assessed using a traction meter consisting of a horizontal limb mesh (BrainScience Idea Co., Ltd., Japan). Three successful limb strength measurements within 2 min were recorded. The mean values of the three measurements were utilized as data.

### Measurement of Blood Pressure (BP)

Mice at 14–19 weeks were used for BP measurement. The systolic, mean, and diastolic BP were measured by a programmable sphygmomanometer (BP-98A; Softron, Japan) using tail-cuff method according to the manufacturer’s instructions (Softron, Japan). Unanesthetized mice were introduced into a small holder putted into a thermostatically controlled warming cylinder and maintained at 37°C during measurement. Statistical analysis for comparison of blood pressure was performed by using two-tailed Student’s t test and results are expressed as mean ± S.D.

### RT-PCR and RT-qPCR

Total RNA was isolated from various tissues of wild-type and mNF90 transgenic mice using TRIzol (Invitrogen, USA) and contaminating genomic DNA was removed using DNA-free (Ambion, USA). cDNA was synthesized using SuperScript III reverse transcriptase (Invitrogen, USA) and random hexamer primer according to the manufacture’s instructions (Invitrogen, USA). PCRs were performed on a Verity 96-well Thermal Cycler (Applied Biosystems, USA) using a cycling program and all runs included hypoxanthine phosphoribosyltransferase (HPRT) gene as an internal control. The PCR products were separated in a 2% agarose gel and visualized by ethidium bromide staining. For RT-quantitative PCR (RT-qPCR), the PCR sample was comprised of diluted cDNA (1/10), SYBR green PCR master mix (Applied Biosystems), and 0.5 µM each of forward and reverse primers in a total volume of 10 µl. PCRs were performed on a StepOne Plus (Applied Biosystems) using a cycling program. All runs included the HPRT gene as an internal control. Samples were normalized to HPRT RNA, giving arbitrary values representing a ratio of experimental to control results. Results were expressed as relative mRNA levels.

### Histological Analysis

Heart and skeletal muscle tissues were fixed with 10% phosphate-buffered formalin and embedded in paraffin. Sections were stained with haematoxylin and eosin and observed under a light microscope.

### Electron Microscopy

Mouse skeletal and cardiac muscles were fixed with 2% glutaraldehyde in 0.1 M phosphate buffer, pH 7.3 for 2 h at 4°C. The tissues were then postfixed with 1% osmium tetroxide (or osmic acid) in 0.1 M phosphate buffer, pH 7.3 for 1 h at 4°C and dehydrated in a graded series of ethanol. Following dehydration, the specimens were transferred to propylene oxide and embedded in Epon 812 (TAAB Laboratories Equipment, Berkshire, England). They were observed with a Hitachi H-7100 electron microscopy (Hitachi, Japan).

### Cell Culture

Human embryonic kidney (HEK) 293 cells (ATCC) were maintained in Dulbecco’s modified Eagle’s medium supplemented with 10% fetal calf serum. Medium containing 4500 mg/l glucose was used unless otherwise stated in figure legends. To generate NF90 stable cell lines, HEK293 cells were transfected with pcDNA3-Flag-NF90b and neomycin was added to the medium to a final concentration of 400 mg/ml at 24 h post transfection. Resistant cells were clonally selected 14 days later and screened by immunoblotting. The selected stable clones were maintained in the presence of neomycin.

### Isolation of NF90-associated Complex

HEK293 cells or stable Flag-NF90b-HEK293 cells were lysed in buffer A (10 mM Hepes-KOH, pH 7.8, 10 mM KCl, 0.1 mM EDTA, 0.1% NP-40, 1 mM DTT, 0.5 mM PMSF) including 1 X protease inhibitor (Roche, Switzerland) by vigorously vortexing for 15 sec. After centrifugation at 10,000 rpm for 5 min at 4°C, the pellets were resuspended in buffer B (50 mM Hepes-KOH, pH 7.8, 420 mM KCl, 0.1 mM EDTA, 5 mM MgCl_2_, 2% glycerol, 1 mM DTT, 0.5 mM PMSF) including 1 X protease inhibitor (Roche), followed by incubation on ice for 30 min. The supernatants were collected as nuclear extracts after centrifugation at 15,000 rpm for 15 min at 4°C. The nuclear extracts were incubated with anti-Flag M2-agarose beads (Sigma, USA) by gently mixing overnight at 4°C to immunoprecipitate the NF90-associated complex. After washing the agarose five times with washing buffer (20 mM Tris-HCl, pH 8.0, 150 mM NaCl, 0.05% Tween 20), the bound complexes were eluted with SDS-PAGE sample buffer. The isolated complexes were analyzed by SDS-PAGE on 15% gels.

### Protein Identification by Peptide Mass Fingerprinting

SDS-PAGE gel fragments containing polypeptides were excised and subjected to in-gel tryptic digestion as described [32]. Peptides generated by the digestion were recovered as described previously [32] and analyzed for their peptide mass fingerprint using MALDI-TOF-MS/MS (AB SCIEX, USA). For protein identification, the peptide masses were examined using the Mascot search engine (Matrix Science Ltd., UK).

### Preparation of Primary Cultures of Mouse Skeletal Muscle

Primary cultures of skeletal muscle from mice were prepared as described previously [33] with some modification. Briefly, the hindlimbs were removed from mice and the bones were dissected away. The remaining muscle mass was weighed, and then minced in a few drops of PBS. Cells were enzymatically dissociated by the addition of approximately 4 ml per g of a solution of 1 mg/ml collagenase type IA (Sigma, USA) supplemented with 2.5 mM CaCl_2_. The slurry was incubated at 37°C for 60 min, mixed by pipetting every 15 min, and then passed through a 40-mm filter. The filtrate was centrifuged at 1,500 rpm for 5 min to sediment the dissociated cells. The pellet was resuspended in Ham’s F-10 nutrient mixture (GIBCO, USA) supplemented with 20% FCS and 1 ng/ml bFGF (ReproCELL, Japan), and the suspension was plated on collagen-coated dishes.

### Measurement of the Protein Synthesis Rate

Cells were cultured for 2 h at 37°C in 5% CO_2_ in methionine free-DMEM (Invitrogen, USA) including [^3^H]methionine. After collecting the cells, they were suspended in a 10% trichloroacetic acid (TCA) solution, followed by incubation on ice for 10 min. To recover the TCA-precipitated protein, the TCA solution was filtered through a glass filter using an aspirator. Radioactivity was measured on the glass filter by liquid scintillation (TRI-CARB 2500TR, PACKARD, USA). The DNA amounts of cells used in this analysis were quantitated by the DABA method [34]. All data were normalized with the DNA amount of the cells in culture on the day of the experiment.

### Western Blot Analysis

Western blot analysis was performed as previously describe [35]. Antibodies were obtained from the following sources: anti-LC3A, anti-LC3B (Cell Signaling Technology), anti-PGC-1α/β, anti-NRF-1 (abcam) and anti-α-tubulin (Calbiochem).

## Supporting Information

Figure S1 **The expression of NF90 in transgenic mice.** (A) Total RNAs isolated from various tissues of wild-type (WT) and NF90 Tg mice (lines TG1 and 2) were analyzed by RT-PCR with specific primers for mouse NF90 or hypoxanthine phosphoribosyltransferase (HPRT). HPRT was used as an internal control. W, wild-type; T1, NF90 Tg mice (line TG1); T2, NF90 Tg mice (line TG2). (B) Immunoblot analysis of NF90 in heart and skeletal muscle of WT and NF90 Tg mice (lines TG1 and 2). Anti-α-tubulin was used as loading control. W, wild-type; T1, NF90 Tg mice (line TG1); T2, NF90 Tg mice (line TG2).
(TIFF)

Figure S2 **Immunohistochemical detection of NF90 in skeletal and cardiac muscle from WT and NF90 Tg mice (line TG1).** Paraffin-embedded tissue sections were prepared and immunostained with anti-mouse-NF90 (III, IV, VII and VIII) or control IgG (I, II, V and VI). The specimens were lightly stained with hematoxylin. Scale bars show 50 mm at the inset.
(TIFF)

Figure S3 **Measurement of plasma catecholamine levels in WT and NF90 Tg mice (line TG1) at 15 weeks to 18 weeks of age.** (A and B) The concentrations of noradrenaline and adrenaline in the plasma of mice are shown in A and B, respectively. All data are expressed as means±SD (n = 9 per group). *, p < 0.01 relative to WT by a two-tailed Student’s t test.
(TIFF)

Figure S4 **HE-stained sections of cardiac muscle from WT (I to V) and NF90 Tg mice (line TG1) (VI to X) at the age of 6 weeks through 10 weeks.** Arrows highlight vacuolations. Scale bars show 10 mm at the inset.
(TIFF)

Figure S5 **Immunoblot analysis of NF90 in cardiac muscle from WT (n = 2) and NF90 Tg mice (line TG1) (n = 2) at the age of 6 weeks through 10 weeks, and at 18weeks of age.** Anti-α-tubulin was used as loading control. W1 and W2, wild-type; T1-1 and -2, NF90 Tg mice (line TG1). Intensities of specific bands in the immunoblotting analysis were measured with a densitometer and are presented as a graph.
(TIFF)

Figure S6 **Immunoblot analysis of caspase-3 and caspase-6 in the skeletal muscles from WT and NF90 Tg mice (lines TG1).** Anti-α-tubulin was used as loading control. W-1 to -6, WT; T-1 to -4, NF90 Tg mice (line TG1).
(TIFF)

Figure S7 **Immunoblot analysis of NF90 in primary cells of skeletal muscles from WT and NF90 Tg mice (lines TG1).** Anti-α-tubulin was used as loading control. W-1 and -2, wild-type; T1-1, -2 and -3, NF90 Tg mice (line TG1).
(TIFF)

Table S1 **A list of NF90-associated proteins.** Molecular weights (MW), NCBI accession numbers (accession No.), cellular localizations and known functions in mammal of the proteins shown in figure 5A are indicated. The known functions in mammals were extracted from the NCBI database.
(XLS)

Table S2 **A list of oligonucleotides used in this study.**
(DOCX)

Methods S1 Detailed procedures and any associated references related to supplementary analyses are described in Methods S1.
(DOCX)
