# Supplementary figures and images for: High Expression of Nuclear Factor 90 (NF90) Leads to Mitochondrial Degradation in Skeletal and Cardiac Muscles
Source: PLoS One. 2012 Aug 17;7(8):e43340. doi: 10.1371/journal.pone.0043340 (PMC3422296; doi:10.1371/journal.pone.0043340)

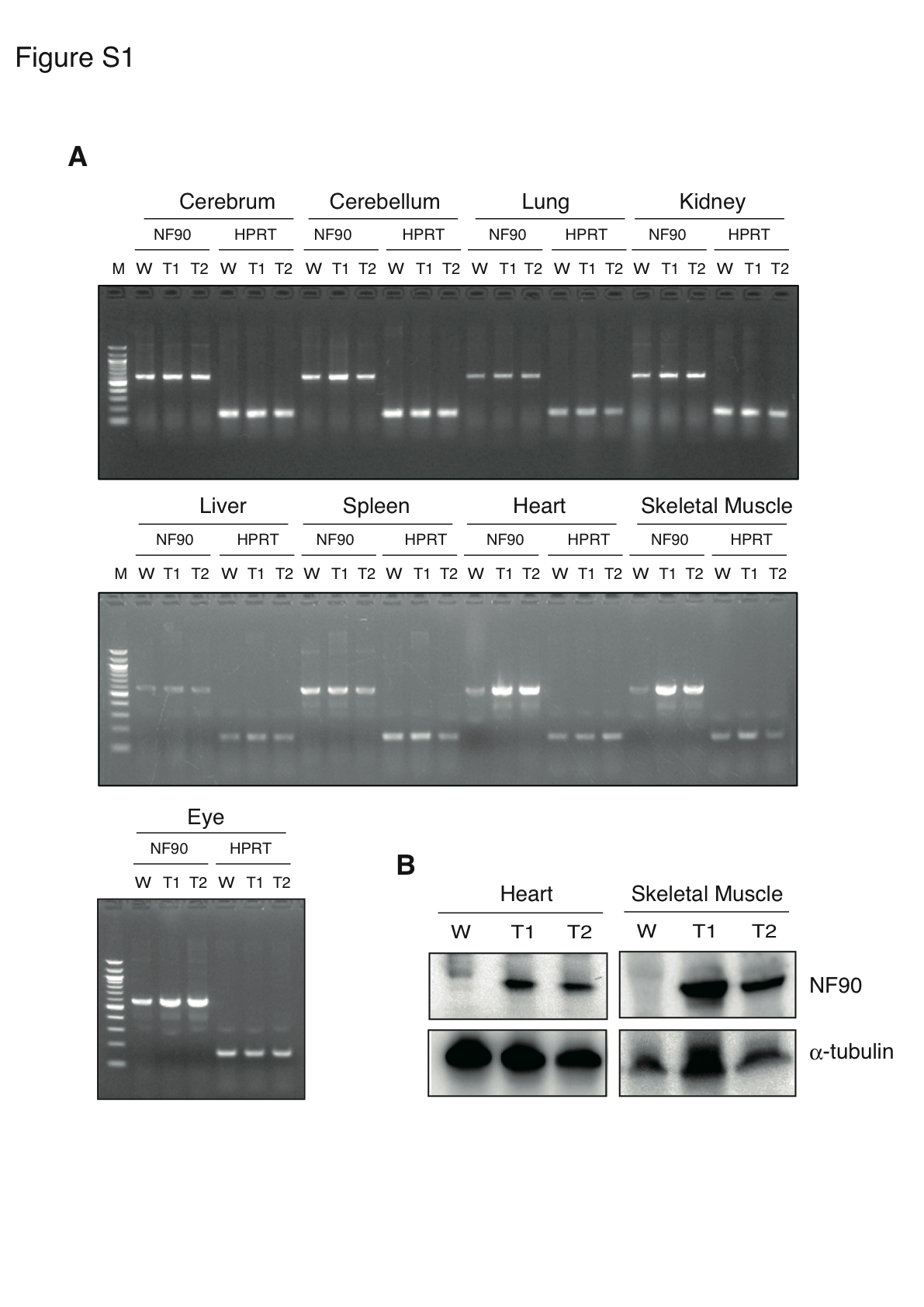

Supplement: Figure S1 — The expression of NF90 in transgenic mice. (A) Total RNAs isolated from various tissues of wild-type (WT) and NF90 Tg mice (lines TG1 and 2) were analyzed by RT-PCR with specific primers for mouse NF90 or hypoxanthine phosphoribosyltransferase (HPRT). HPRT was used as an internal control. W, wild-type; T1, NF90 Tg mice (line TG1); T2, NF90 Tg mice (line TG2). (B) Immunoblot analysis of NF90 in heart and skeletal muscle of WT and NF90 Tg mice (lines TG1 and 2). Anti-α-tubulin was used as loading control. W, wild-type; T1, NF90 Tg mice (line TG1); T2, NF90 Tg mice (line TG2). (TIFF) [file pone.0043340.s001.tiff]

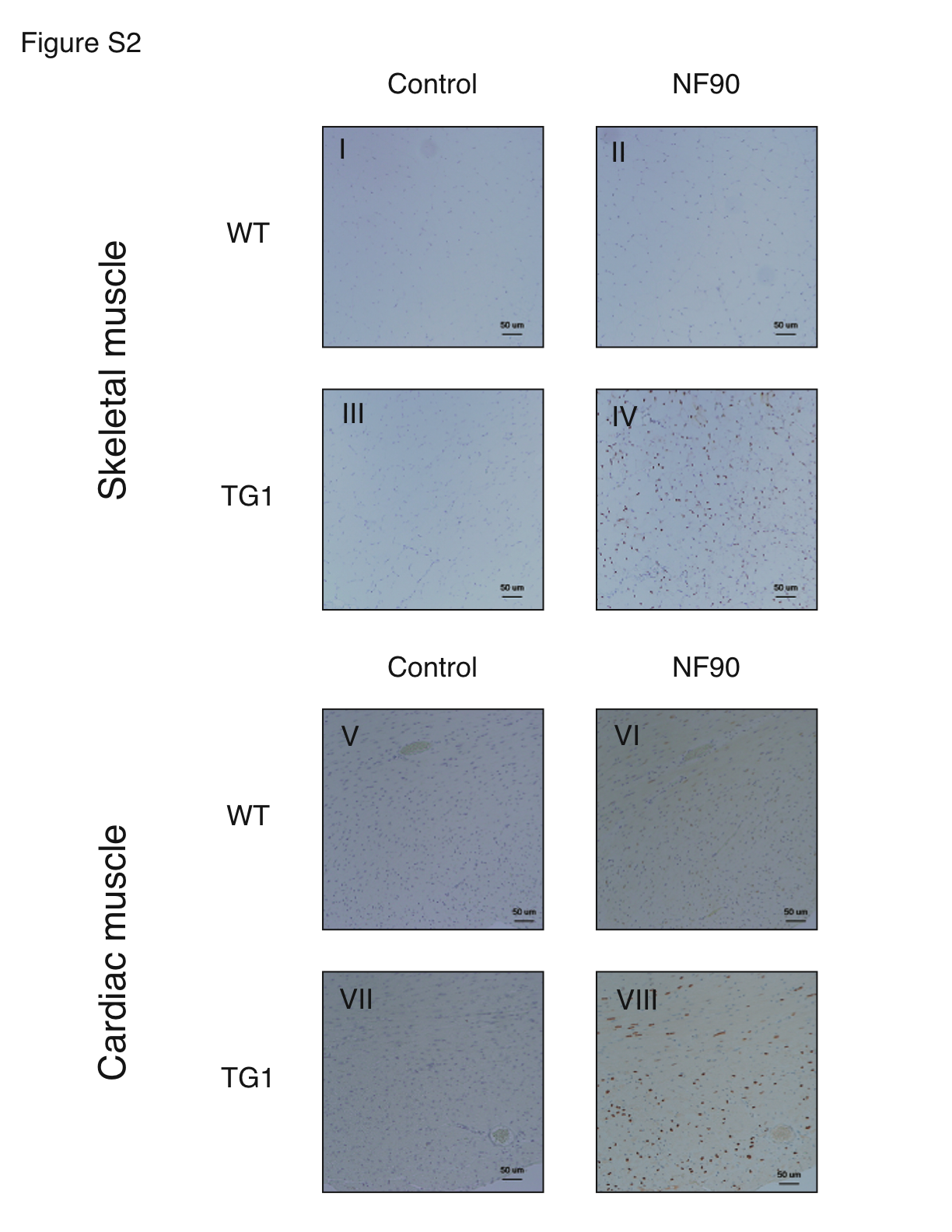

Supplement: Figure S2 — Immunohistochemical detection of NF90 in skeletal and cardiac muscle from WT and NF90 Tg mice (line TG1). Paraffin-embedded tissue sections were prepared and immunostained with anti-mouse-NF90 (III, IV, VII and VIII) or control IgG (I, II, V and VI). The specimens were lightly stained with hematoxylin. Scale bars show 50 mm at the inset. (TIFF) [file pone.0043340.s002.tiff]

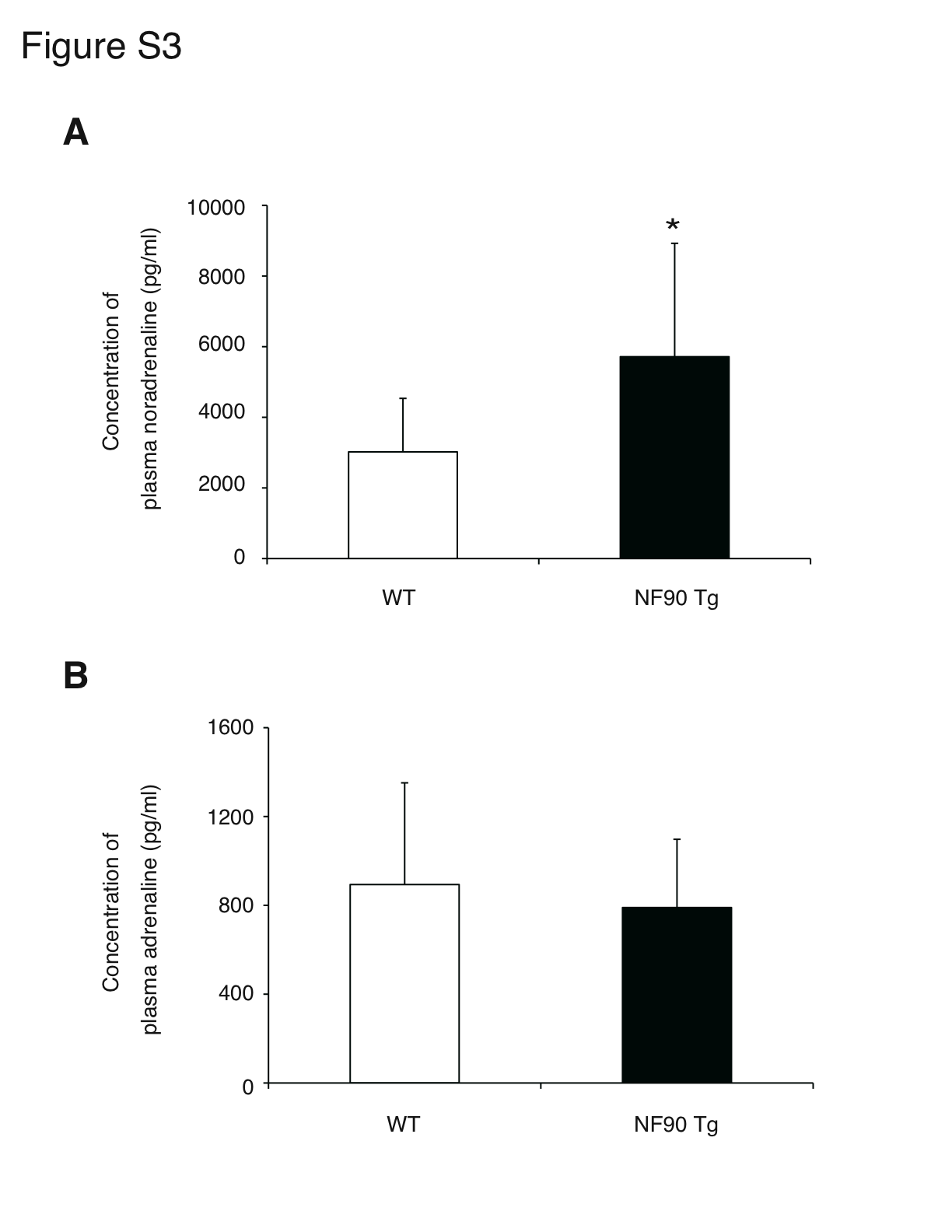

Supplement: Figure S3 — Measurement of plasma catecholamine levels in WT and NF90 Tg mice (line TG1) at 15 weeks to 18 weeks of age. (A and B) The concentrations of noradrenaline and adrenaline in the plasma of mice are shown in A and B, respectively. All data are expressed as means±SD (n = 9 per group). *, p < 0.01 relative to WT by a two-tailed Student’s t test. (TIFF) [file pone.0043340.s003.tiff]

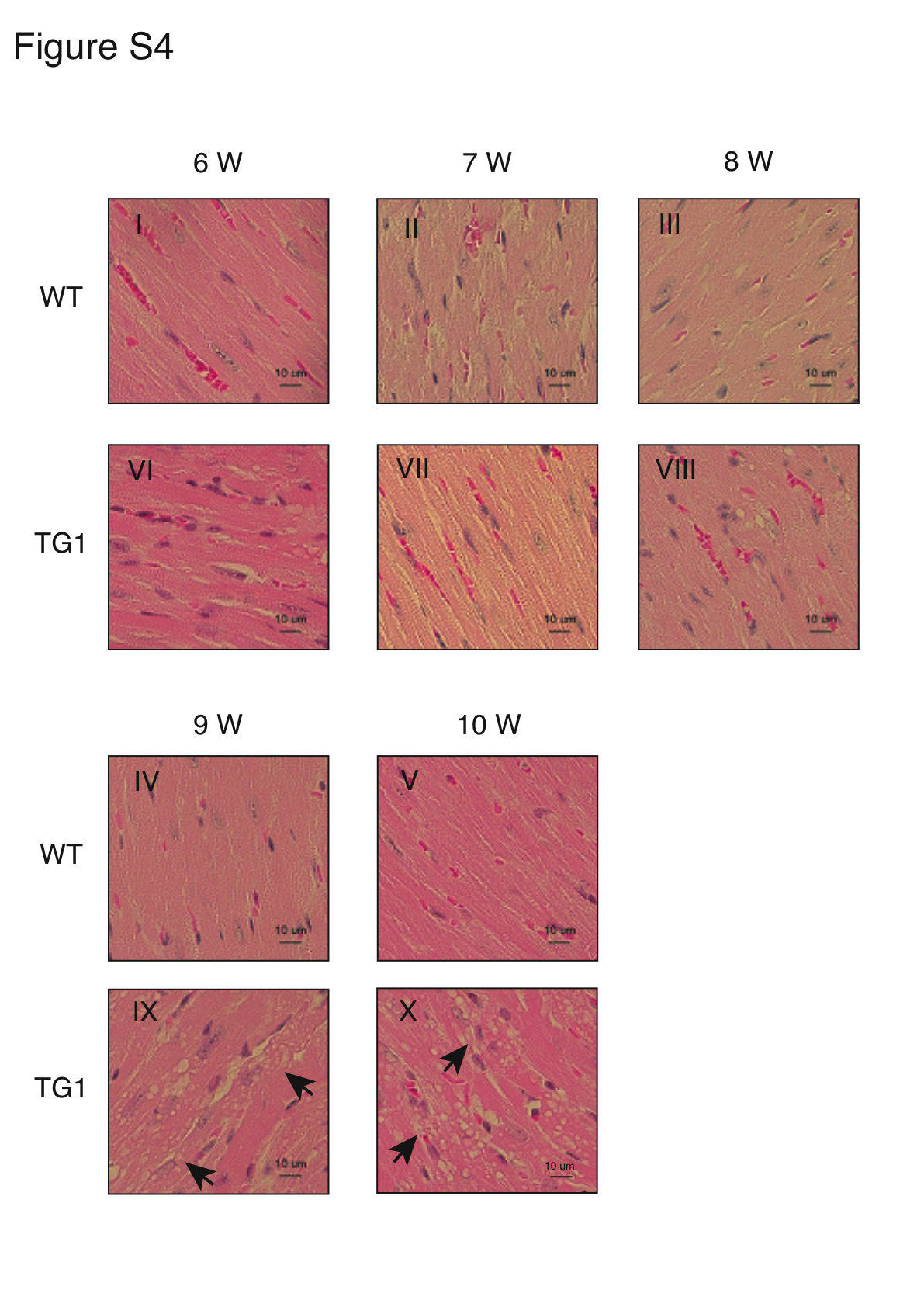

Supplement: Figure S4 — HE-stained sections of cardiac muscle from WT (I to V) and NF90 Tg mice (line TG1) (VI to X) at the age of 6 weeks through 10 weeks. Arrows highlight vacuolations. Scale bars show 10 mm at the inset. (TIFF) [file pone.0043340.s004.tiff]

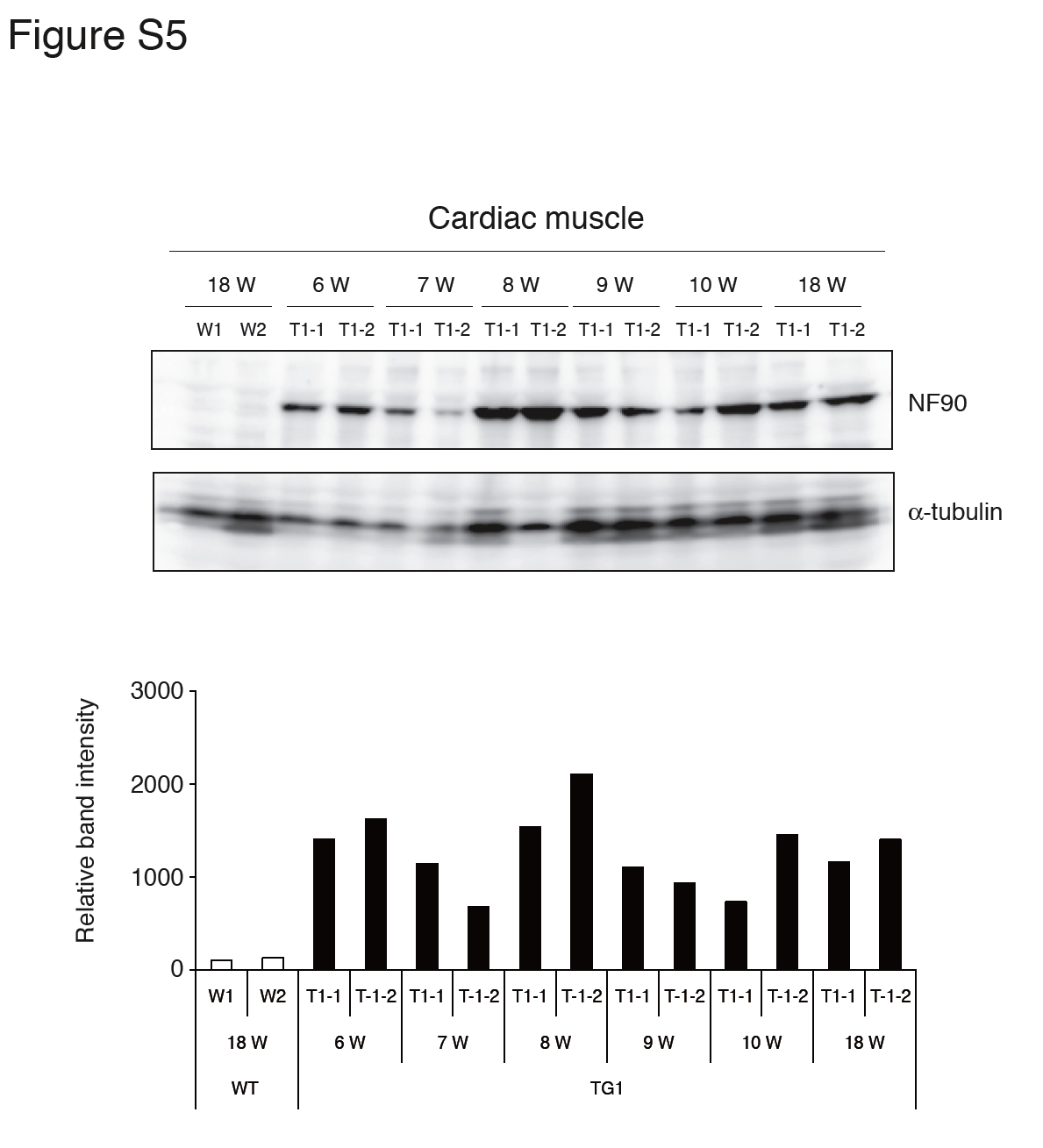

Supplement: Figure S5 — Immunoblot analysis of NF90 in cardiac muscle from WT (n = 2) and NF90 Tg mice (line TG1) (n = 2) at the age of 6 weeks through 10 weeks, and at 18weeks of age. Anti-α-tubulin was used as loading control. W1 and W2, wild-type; T1-1 and -2, NF90 Tg mice (line TG1). Intensities of specific bands in the immunoblotting analysis were measured with a densitometer and are presented as a graph. (TIFF) [file pone.0043340.s005.tiff]

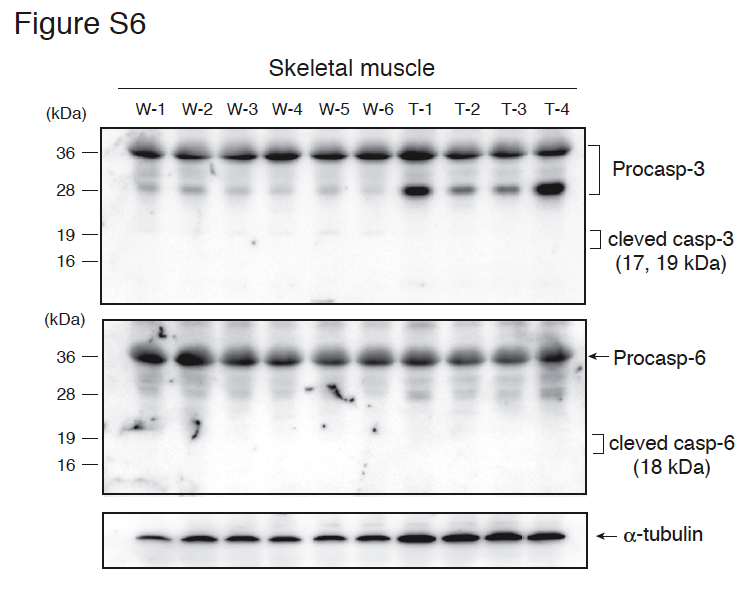

Supplement: Figure S6 — Immunoblot analysis of caspase-3 and caspase-6 in the skeletal muscles from WT and NF90 Tg mice (lines TG1). Anti-α-tubulin was used as loading control. W-1 to -6, WT; T-1 to -4, NF90 Tg mice (line TG1). (TIFF) [file pone.0043340.s006.tiff]

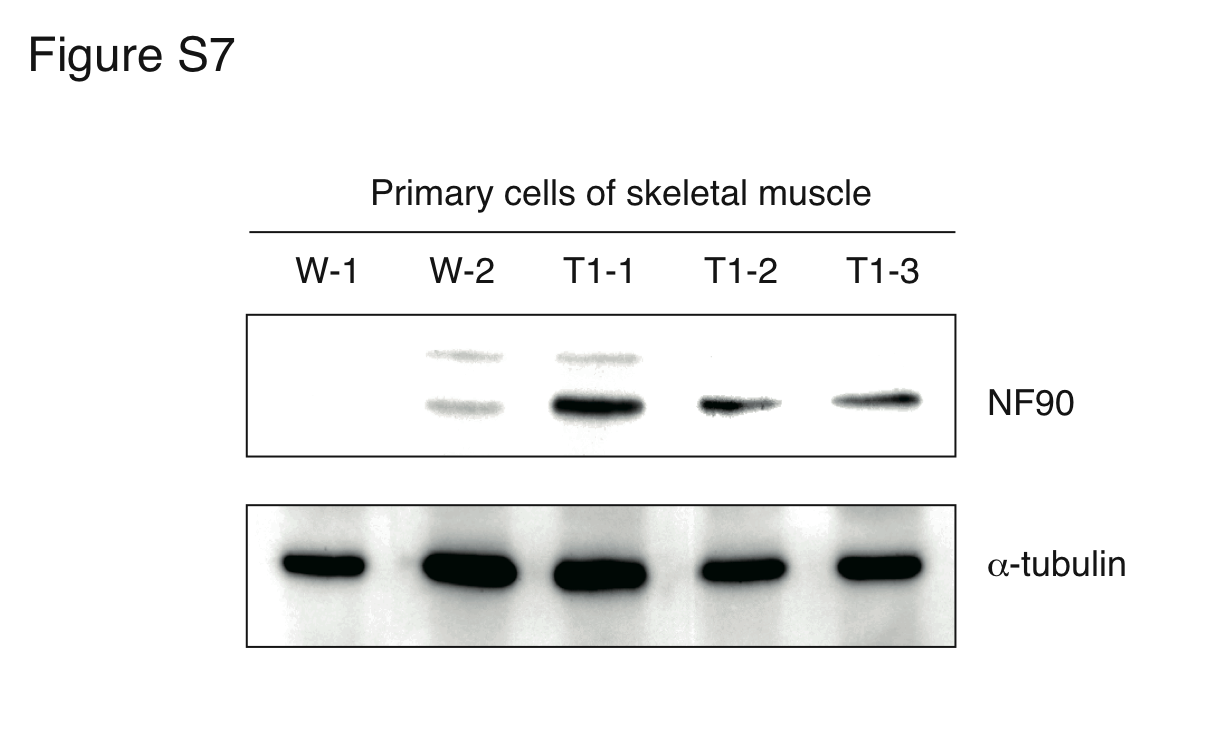

Supplement: Figure S7 — Immunoblot analysis of NF90 in primary cells of skeletal muscles from WT and NF90 Tg mice (lines TG1). Anti-α-tubulin was used as loading control. W-1 and -2, wild-type; T1-1, -2 and -3, NF90 Tg mice (line TG1). (TIFF) [file pone.0043340.s007.tiff]
