# Supplementary material for: High Expression of Nuclear Factor 90 (NF90) Leads to Mitochondrial Degradation in Skeletal and Cardiac Muscles
Source: PLoS One. 2012 Aug 17;7(8):e43340. doi: 10.1371/journal.pone.0043340 (PMC3422296; doi:10.1371/journal.pone.0043340)
Supplement: Table S2 — A list of oligonucleotides used in this study. (DOCX) [file pone.0043340.s009.docx]

**Table S2. Oligonucleotides Used in This Study**

Oligonucleotides 　　 Oligonucleotides sequence (5’ to 3’)　　 Reference

**Cloning for expression plasmids**

NF90 F CCGCTCGAGATGGCATTGTATCATCATCA In this study

NF90R CCGCTCGAGTTGGGTTTTGGAGTTGG In this study

**RT-PCR**

NF90 F TGCCCAAGAAACCAAAGA In this study

NF90 R TTGAGGCCACGTCTCTTC In this study

HPRT F GTGGCCCTCTGTGTGCTGAA In this study

HPRT R TTATGTCCCCCGTTGACTGGTC In this study

BNP F CTGAAGGTGCTGTCCCAGATG [[1](#_ENREF_1)]

BNP R GACGGATCCGATCCGGTC [[1](#_ENREF_1)]

PGC-1α F CGGAAATCATATCCAACCAG [[2](#_ENREF_2)]

PGC-1α R TGAGAACCGCTAGCAAGTTTG [[2](#_ENREF_2)]

PGC-1β F GGACGCCAGTGACTTTGACT [[3](#_ENREF_3)]

PGC-1β R TTCATCCAGTTCTGGGAAGG [[3](#_ENREF_3)]

COX-2 F CCGACTAAATCAAGCAACAGTAACA [[4](#_ENREF_4)]

COX-2 R AAATTTCAGAGCATTGGCCATAG [[4](#_ENREF_4)]

COX-4 F CTATGTGTATGGCCCCATCC [[4](#_ENREF_4)]

COX-4 R AGCGGGCTCTCACTTCTTC [[4](#_ENREF_4)]

NRF-1 F CCACGTTGGATGAGTACACG [[5](#_ENREF_5)]

NRF-1 R CTGAGCCTGGGTCATTTTGT [[5](#_ENREF_5)]

**References**

1. Abdelalim EM, Tooyama I (2009) BNP signaling is crucial for embryonic stem cell proliferation. PLoS One 4: e5341.

2. Miura S, Kawanaka K, Kai Y, Tamura M, Goto M, et al. (2007) An increase in murine skeletal muscle peroxisome proliferator-activated receptor-gamma coactivator-1alpha (PGC-1alpha) mRNA in response to exercise is mediated by beta-adrenergic receptor activation. Endocrinology 148: 3441-3448.

3. Sahin E, Colla S, Liesa M, Moslehi J, Muller FL, et al. (2011) Telomere dysfunction induces metabolic and mitochondrial compromise. Nature 470: 359-365.

4. Miura S, Kai Y, Kamei Y, Ezaki O (2008) Isoform-specific increases in murine skeletal muscle peroxisome proliferator-activated receptor-gamma coactivator-1alpha (PGC-1alpha) mRNA in response to beta2-adrenergic receptor activation and exercise. Endocrinology 149: 4527-4533.

5. Casas F, Pessemesse L, Grandemange S, Seyer P, Gueguen N, et al. (2008) Overexpression of the mitochondrial T3 receptor p43 induces a shift in skeletal muscle fiber types. PLoS One 3: e2501.
