# Supplementary material for: High Expression of Nuclear Factor 90 (NF90) Leads to Mitochondrial Degradation in Skeletal and Cardiac Muscles
Source: PLoS One. 2012 Aug 17;7(8):e43340. doi: 10.1371/journal.pone.0043340 (PMC3422296; doi:10.1371/journal.pone.0043340)
Supplement: Methods S1 — Detailed procedures and any associated references related to supplementary analyses are described in Methods S1. (DOCX) [file pone.0043340.s010.docx]

**Supplementary Methods S1**

*RT-PCR.* Total RNA was isolated from various tissues of wild-type and mNF90 transgenic mice using TRIzol (Invitrogen, USA) and contaminating genomic DNA was removed using DNA-free (Ambion, USA). cDNA was synthesized using SuperScript III reverse transcriptase (Invitrogen, USA) and random hexamer primer according to the manufacture’s instructions (Invitrogen, USA). PCRs were performed on a Verity 96-well Thermal Cycler (Applied Biosystems, USA) using a cycling program and all runs included hypoxanthine phosphoribosyltransferase (HPRT) gene as an internal control. The PCR products were separated in a 2% agarose gel and visualized by ethidium bromide staining.

*Western blot analysis.* Western blot analysis was performed as previously describe [[1](#_ENREF_1)].

*Antibody.* An anti-mouse NF90 antibody was produced by immunizing New Zealand White rabbits with recombinant His-mouse NF90 (residues 334 to 716) as previously described [[2](#_ENREF_2)]. Anti-Caspase-3 and anti-Caspase-6 were obtained from Cell Signaling Technology. Anti-α-tubulin was obtained from CalBiochem.

*Immunohistochemistry.* Heart and skeletal muscle tissues were fixed with 10% phosphate-buffered formalin and embedded in paraffin, and sectioned. The sections were deparaffined, rehydrated, followed by boiling in citrate buffer, pH 6.0 for 20 min to retrieve antigen. After quenching of hydrogen peroxide, the specimens were incubated with 1:1000 dilution of anti-mouseNF90 and were then stained with Histofine Simple Stain MAX PO (MULTI) (universal immuno-peroxidase polymer, anti-mouse and –rabbit) according to the manufacturer’s instructions (Nichirei Biosciences Inc., Japan).

*Measurement of plasma catecholamines.* Plasma catecholamines were measured as previously described [[3](#_ENREF_3)].

*Histological analysis.* Cardiac muscle tissues were fixed with 10% phosphate-buffered formalin and embedded in paraffin. Sections were stained with haematoxylin and eosin and observed under a light microscope.

**References**

1. Sakamoto S, Taniguchi T (2001) Identification of a phorbol ester-responsive element in the interferon-gamma receptor 1 chain gene. J Biol Chem 276: 37237-37241.

2. Song D, Sakamoto S, Taniguchi T (2002) Inhibition of poly(ADP-ribose) polymerase activity by Bcl-2 in association with the ribosomal protein S3a. Biochemistry 41: 929-934.

3. Okada S, Yamaguchi N (2010) Alpha1-adrenoceptor activation is involved in the central N-methyl-D-aspartate-induced adrenomedullary outflow in rats. Eur J Pharmacol. Netherlands: 2010 Elsevier B.V. pp. 55-62.
